# Angiotensinogen M235T gene variants and its association with essential hypertension and plasma renin activity in Malaysian subjects: A case control study

**DOI:** 10.1186/1471-2261-5-7

**Published:** 2005-04-05

**Authors:** Yee-How Say, King-Hwa Ling, Gnanasothie Duraisamy, Suzanne Isaac, Rozita Rosli

**Affiliations:** 1 Department of Human Growth and Development, Faculty of Medicine and Health Sciences, Universiti Putra Malaysia, 43400 UPM Serdang, Selangor DE, Malaysia; 2 Department of Clinical Laboratory Sciences, Faculty of Medicine and Health Sciences, Universiti Putra Malaysia, 43400 UPM Serdang, Selangor DE, Malaysia; 3 Health Clinic, Kuala Lumpur Hospital, Jalan Pahang, 50588 Kuala Lumpur, Malaysia

## Abstract

**Background:**

Essential hypertension is a major public health concern worldwide where its prevalence accounts for various cerebrovascular diseases. A common molecular variant of angiotensinogen (AGT), the precursor of potent vasoactive hormone angiotensin II, has been incriminated as a marker for genetic predisposition to essential hypertension in some ethnics. This case-control study was designed not only to determine the association of the AGT M235T gene variants with essential hypertension, but also its relationship to Plasma Renin Activity (PRA) in subjects attending the Health Clinic, Kuala Lumpur, Malaysia.

**Methods:**

The study involved 188 subjects, 101 hypertensives and 87 normotensives. Consents were obtained from all the participated subjects. M235T gene variants were investigated using allele specific polymerase chain reaction and PRA was determined by radioimmunoassay. Hypertensinogenic factors such as dietary habits, physical activity, smoking and drinking habits were assessed using a pre-tested questionnaire.

**Results:**

The genotype and allele distribution of the M235T variant differed significantly in hypertensives and normotensives (χ^2 = ^23.184, P < 0.001 and χ^2 ^= 21.482, P < 0.001, respectively). The odds ratio for hypertension was 1.36 (95% confidence interval 1.03–1.80) for subjects with homozygous mutated allele TT of the M235T variant compared with other genotypes or 1.98 (95% confidence interval 1.46–2.67) for those carrying T allele compared to those carrying M allele. Plasma Renin Activity is also significantly higher in hypertensive subjects (PRA = 3.8 ± 2.5 ngAI/ml/hr for hypertensives, PRA = 2.6 ± 1.3 ngAI/ml/hr for normotensives, P < 0.001), but was not significantly different between groups of genotypes (P = 0.118).

**Conclusion:**

The M235T variant of the AGT is significantly associated with essential hypertension whereas the genotype TT or allele T is a possible genetic marker or risk factor for hypertension in Malaysian subjects.

## Background

High blood pressure or hypertension, 'a silent-killer' condition, is now the most common chronic condition, affecting 20–30% of the adult population. It is rapidly becoming a major problem in developing countries, including Malaysia. About 90–95% of hypertension (HTN) is idiopathic and apparently primary or essential hypertension (EH). Of the remaining 5–10%, most are secondary to renal and adrenal diseases. EH is a multifactorial disorder arising from the influence of several susceptibility genes and environmental stimuli. Evidence suggests that genes may contribute to 30% of the variation of blood pressure. However, the number of genes involved or the model of interaction with other genes or environmental risk factors is unknown.

The angiotensiongen (AGT) gene regulates the expression of angiotensinogen, a polypeptide primarily produced by the liver. Cleavage of the angiotensinogen molecule by renin, liberating angiotensin I, and then converted into angiotensin II by angiotensin-converting enzyme. This product binds to its receptor, exerting physiologic effects on the sodium homoeostasis and vascular resistance, thus regulates the blood pressure [1]. The plasma concentration of AGT is correlated with blood pressure [1,2]. Mice with the AGT gene duplicated have blood pressure and plasma AGT levels positively correlated with the number of gene copies [3].

The human AGT gene has been cloned and sequenced [4]. Fifteen molecular variants have been identified and only three have so far been reported to have a possible genetic association with hypertension [5]. One variant encodes threonine instead of methionine at position 235(T235) [5,6], the others encode methionine instead of threonine at position 174 [5,6] and the microsatellite (a GT-repeat sequence, varies highly as 11 different allelic variants among individuals) [7]. Association studies of the M235T variant in essential hypertension have yielded conflicting results. Some found linkage or association in Caucasian [5-7], African-Carribean [8], Japanese [9,10] and Taiwanese populations [11], while others did not [12-14]. A molecular variant of AGT has also been reported to be associated with preeclampsia [15].

The study addressed the question as to whether there is an association between the AGT M235T gene variant and essential hypertension in the Malaysian subjects since genetic diversity exists among different ethnic populations and realizing the fact that the association in one population could not be extrapolated to another population.

## Methods

### Study subjects

Approval and permission were obtained from the ethics committees of the Faculty of Medicine and Health Sciences, Universiti Putra Malaysia, the Federal Territory Kuala Lumpur Health Department and the Ministry of Health, Malaysia to meet the ethics guidelines. The permission allowed the study to be carried out in the Health Clinic, Kuala Lumpur Hospital. Upon the approval, subjects were recruited consecutively from patients attending the Health Clinic from 1^st ^October to 31^st ^October, 2002. The patients referred to the clinic were residents of the Klang Valley, consisting of the Federal Territory Kuala Lumpur and parts of the Selangor state. The subjects can be categorized into three main ethnic groups: Malay, Chinese and Indian. Hypertensive subjects were defined as those with systolic blood pressure (SBP) of greater than or equal to 140 mmHg, with a diastolic blood pressure (DBP) of greater than or equal to 90 mmHg, or are currently administered at least one hypertensive medication. Any subjects with the possibility of secondary hypertension were excluded. Hypertensive subjects whose parents both had hypertension were considered to have a positive family history of hypertension. Normotensive was defined as those with a blood pressure of less than 140/90 mmHg; those with a positive family history of hypertension were excluded. Both groups with subjects under the influence of estrogen, thyroid and cortisol hormones were excluded. The subjects were selected by medical officers and also approached by the field team. Informed consent was obtained from the subjects and a total of 101 hypertensives (23 males and 68 females) and 87 normotensives (23 males and 64 females) were recruited.

### Questionnaire, blood pressure and body mass index measurements

A three-page pre-tested questionnaire in both the Malay and English language were developed to assess the socio-demographic background, socio-demographic data, dietary habits, physical activity, smoking habits, alcohol consumption and family history for hypertension. The blood pressure (BP) was measured with the subject sitting, using an automated sphygmomanometer (Colin Press-Mate BP-8800C^®^) after at least 10 minutes of resting. Height and weight of subjects were obtained by using the TANITA digital weighing scale and the SECA Bodymeter 208 respectively. The body mass index (BMI) of subjects was calculated as weight (kg) / height^2 ^(m^2^).

### Blood collection

Four to five mililitres of peripheral venous blood were collected into two separate K_2 _EDTA vacutainer test tubes from each subject for radioimmunoassay (RIA) and AGT M235T variant genotyping. Overnight fasting blood samples were collected into tubes containing sodium fluoride. Blood was collected using a 21-Gauge needle with a 5.0 ml syringe by a qualified phlebotomist. The collected blood in test tubes were kept at 4°C and centrifuged at 1200 g for 15 minutes in order to separate the plasma from whole blood. The plasma samples were frozen at -20°C until fasting blood glucose (FBG), triglycerides (TG), total cholesterol (TC), low-density lipoprotein (LDL-C), high density lipoprotein (HDL-C) and its percentage, plasma sodium and potassium levels were determined within 3 days.

### Detection of the AGT genotypes

Genomic DNA extraction was carried out using the QIAamp^® ^DNA Blood Mini Kit by QIAGEN^®^. DNA fragments including the M235T variant were amplified by allele-specific polymerase chain reaction (PCR). The AGT M235T polymorphism was typed by the previously described mismatch priming method with some modifications [12]. The forward primer sequence from +921 to +941 in exon 2 of the AGT gene is 5' GAT GCG CAC AAG GTC CTG TC 3' whereas the reverse primer sequence from +1202 to +1224 is 5' GGT GCT GTC CAC ACT GGA CCC C 3'. The reverse primer was designed to contain a base substitution T→C at the fourth last nucleotide from its 3' end [12]. The individual PCR reaction vial contains a final volume of 50 μl solution. One-hundred nanograms of DNA samples was added to 14 μl of PCR master mix consisting of 5.0 μl of Promega^® ^10X Mg Free PCR Buffer, 3.0 μl of Promega^® ^25 mM MgCl_2_, 2.5 μl of 10 pM forward primer, 2.5 μl of 10 pM reverse primer and 1.0 ml of Promega^® ^10 mM dNTP. An appropriate amount of sterile ultrapure water (which totals up to 50 μl) was added to each of the microfuge tube. One microlitre of 5 units/μl *Taq *DNA polymerase was added to the reaction vial only after 5 minutes of pre-denaturation process prior to performing 'hot start' PCR. The 10X Mg-free PCR Buffer has a composition of 50 mM KCl, 10 mM Tris-HCl, (pH 9.0 at 25°C) and 0.1% Triton^® ^X-100. The PCR was performed using the Mastercycler Gradient (Eppendorf^®^) for 35 cycles. The temperature for the initial denaturation of DNA was 95°C for 1 minute, annealing at 71°C for 1 minute and extension 72°C for 1 minute and a final extension at 72°C for 7 minutes following the last cycle. The PCR product was subjected to *Psy*I (isoschizomere for *Tth111*I restriction enzyme) digestion for 3 hours at 37°C and electrophoresed on a 5.0% agarose gel with ethidium bromide staining.

### Plasma renin activity radioimmunoassay

Plasma renin activity (PRA) was determined using the Angiotensin I [^125^I] RIA kit (PerkinElmer Life Sciences, Inc.). It is a two-step protocol: the generation of Angiotensin I and the RIA to quantitate the amount of Angiotensin I generated. Plasma was incubated at 37°C, at pH 6, with protease inhibitors (EDTA, dimercaprol (BAL) and 8-hydroxyquinoline) for 1 hour to allow the generation of angiotensin I by the endogenous renin and substrate reaction. The total concentration of the Angiotensin I was determined by radioimmunoassay. Plasma renin activity was expressed as ng/ml/hr of angiotensin I generated. The intra-assay coefficient of variation for this assay was 4.0 % (n = 12) and the inter-assay coefficient of variation was 10.4% (n = 84).

### Statistical analysis

The SPSS (previously known as Statistical Package for Social Sciences) for Windows^® ^Version 11.0 was used to statistically analyze the data obtained. Descriptive statistics were used to analyze all variable studies such as the socio-demographic characteristics, dietary patterns, physical activity, smoking practices and alcohol consumption, anthropometric measurements, biological parameters and AGT genotypes of the subjects. Genotype and allele frequencies in control and hypertensive groups were compared by Chi-square (χ^2^) analysis. Continuous variables were compared between hypertensive and control groups by Student's *t *test (or the Mann-Whitney *U *test for non-normally distributed variables). The influence of AGT genotype on continuous variable was investigated by one-way ANOVA. In addition, the effect of AGT genotype on BP was investigated with the General Linear Model ANOVA with adjustment for age, sex, race and BMI. Multiple regression analysis was also performed with SBP or DBP as the dependent variable and sex, age, BMI, total cholesterol and Plasma Renin Activity and AGT genotype (coded 0, 1, or 2 according to the number of T235 alleles) as independent variables. P < 0.05 was considered statistically significant.

## Results

### Demographic data

There were a total of 188 subjects recruited in the study, consisting of 101 hypertensives (33 males and 68 females) and 87 normotensives (23 males and 64 females). Of the 150 subjects approached, the respondence rate was around 72% due to some who choose not to participate or was excluded due to non-compliance of the inclusion criteria. The majority of the subjects were females. The Malays (n = 97, 51.6%) comprises more than half of the subjects, followed by Chinese (n = 56, 29.8%) and Indians (n = 35, 18.6%).

The hypertensive subjects ranged from 30 to 78 years old, with a mean age of 54.7 years, while the normotensives ranged from 25 to 78 years old, with a mean 49.3 years, indicating that the normotensives are younger in the age group. Sixty-three people or 62.4% of the hypertensive subjects have a family history of hypertension while all of the normotensives do not have a family history of hypertension in order to be included in the study.

### Genotypes and allele frequencies

**Figure 1:**
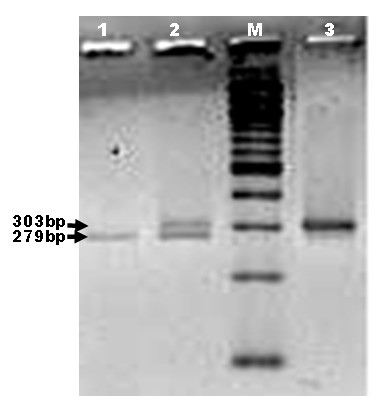
**5.0% agarose gel electrophoresis. **Lanes 1, 2 and 3 correspond to RFLP pattern of homozygous mutant (TT), heterozygous (MT) and homozygous wild-type (MM), respectively. M is a 100 bp linear DNA ladder (Promega).

Figure 1 shows the results of the *PsyI *digestion on PCR products. AGT +704 T→C missense mutation (cause amino acid substitution of AGT M235T) created a new restriction site with the sequence recognition: GACN NNGT↓C for *Psy*I. *Psy*I digested the fragment into 2 parts, the longer fragment, 279 bp and the shorter 24 bp. However, the 5.0% agarose gel was unable to retain the shorter fragment and it was suspected to have migrated out of the gel. Therefore, a band at 303 bp indicates homozygous wild-type (MM), a band at 279 bp indicates homozygous mutated (MT) and two bands at 303 bp and 279 bp indicates heterozygous mutation (TT).

According to Table 1, the prevalence of AGT M235T missense mutation in all the subjects was 17% for homozygous mutation (21% for hypertensives and 11% for normotensives), 34% for heterozygous mutation (46% for hypertensives and 21% for normotensives respectively) and 49% for homozygous wild-type (33% for hypertensives and 68% for normotensives respectively). The allele frequencies and genotype distribution of the M235T variant were in the Hardy-Weinberg equilibrium in either data set for cases (χ^2 ^= 0.634, df = 2, p > 0.5) and for control (χ^2 ^= 917.1, df = 2, p > 0.5) subjects. There was significant difference in genotype and allele frequencies between hypertensive and normotensive groups.

**Table 1:** Genotype and Allele Frequencies of M235T variant of the AGT gene for both cases and controls.

**Group**	**Genotypes**			**Alleles**	
	
	**MM**	**TM**	**TT**	**M**	**T**
**Hypertensives**	33	46	22	112	90
**Normotensives**	59	18	10	136	38

**Total**	92	64	32	248	128

**χ^2^value**	23.184			21.482	
P* value	<0.001			<0.001	

**Odds ratio**	1.36			1.98	

*Significant values were obtained through the Chi-square test.

When the allele frequencies are categorized based on ethnic groups (Table 2), there were significant differences of the prevalence of T allele between hypertensives and normotensives in the Malays (45 controls and 52 cases, χ^2 ^= 12.765, df = 2, p = 0.001) and Indian (20 controls and 15 cases, χ^2 ^= 12.519, df = 2, p = 0.001) but not the Chinese (22 controls and 34 cases, χ^2 ^= 3.083, df = 2, p = 0.212). In addition, there was significance difference between the prevalence of the T allele of hypertensive and normotensive groups in female (23 controls and 33 cases, χ^2 ^= 20.828, df = 2, p = 0.001) but no significant differences between groups in male (64 controls and 68 cases, χ^2 ^= 3.836, df = 2, p = 0.140).

**Table 2:** Frequencies of M235T variant of the AGT gene according to ethnic background for both cases and controls.

**Group**	**Male**		**Female**	
	
	**M**	**T**	**M**	**T**
**Hypertensives**				
Malay	28	14	36	26
Chinese	10	10	28	20
Indian	1	3	9	17

**Total**	39	27	73	63

**Normotensives**				
Malay	25	5	48	12
Chinese	4	4	28	8
Indian	7	1	24	8

**Total**	36	10	100	28

**χ^2^value**	3.836		20.828	

P* value	0.140		0.001	

*Significant values were obtained through the Chi-square test.

### PRA levels

**Figure 2:**
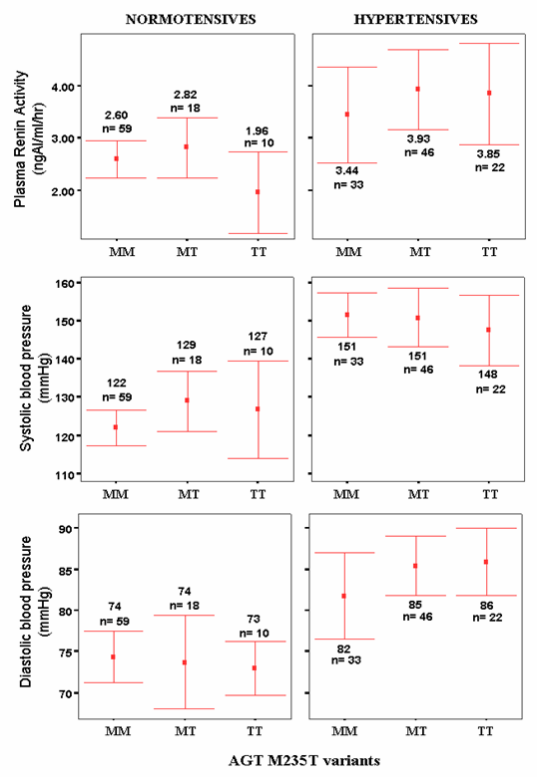
**Plasma renin activity (PRA), systolic blood pressure (SBP) and diastolic blood pressure (DBP) for normotensive and hypertensive subjects as grouped according to AGT M235T genotypes. **The values were expressed as mean (as indicated by the red dots) whereas the bars represent the 95.0% confidence interval of standard deviations. N represents the number of subjects. MM, MT and TT correspond to homozygous wild-type, heterozygous and homozygous mutant, respectively.

According to Table 3, PRA was significantly higher (p < 0.001) in hypertensive subjects (n = 101, 3.8 ± 2.5 ngAI/ml/hr) compared to normotensive subjects (n = 87, 2.6 ± 1.3 ngAI/ml/hr,). However, the PRA was higher in hypertensives group among all the genotypes but not significantly different between genotypes classes (p = 0.687, hypertensives and p = 0.252, normotensives) as shown in Figure 2.

**Table 3:** Plasma renin activity (PRA) levels according to genotypes and genders in both the cases and controls. Values are expressed in ngAI/ml/hr ± standard deviation. Values in parentheses represent the number of subjects.

**Genotypes**	**Normotensives**		**Hypertensives**		**Total**		p*
	
	**Male**	**Female**	**Male**	**Female**	**Male**	**Female**	
**MM**	2.77 ± 1.69 (15)	2.54 ± 1.24 (44)	3.68 ± 2.14 (13)	3.29 ± 2.97 (20)	3.19 ± 1.93 (28)	2.77 ± 1.96 (64)	0.118^a^

**MT**	3.16 ± 1.29 (6)	2.65 ± 1.24 (12)	3.88 ± 2.90 (13)	3.95 ± 2.51 (33)	3.65 ± 2.49 (19)	3.61 ± 2.31 (45)	0.292^e^

**TT**	1.47 ± 1.38 (2)	2.08 ± 1.28 (8)	2.56 ± 1.47 (7)	4.45 ± 2.34 (15)	2.32 ± 1.45 (9)	3.63 ± 2.31 (23)	0.129^f^

**Total**	2.76 ± 1.57 (23)	2.50 ± 1.24 (64)	3.52 ± 2.35 (33)	3.87 ± 2.62 (68)	3.21 ± 2.09 (56)	3.21 ± 2.17 (132)	
	
	2.57 ± 1.32 (87)		3.75 ± 2.53 (101)		3.21 ± 2.14 (188)		

***p*^§^**	0.434^b^		0.521^c^		0.997^d^		
	
	<0.001^g^						

*Significant values were obtained through one-way ANOVA between ^a^groups of genotypes, ^e^among males between groups of genotypes and ^f^among females between groups of genotypes.

^§^Significant values were obtained through Student T-test between ^b^normotensive males and females, ^c^hypertensive males and females, ^d^males and females, and ^g^normotensive and hypertensive subjects.

The means of the PRA were not significantly different between female and male among hypertensives and normotensives. One way ANOVA also showed that there was no significant difference of PRA between age groups in both hypertensives and normotensives (p = 0.611 and p = 0.119, respectively).

### BP variations

Table 4 shows BP and other variables according to AGT genotypes in hypertensive and normotensive data sets. There was no significant difference between genotype classes for both normotensives and hypertensives for unadjusted BP and any other measured variable. After adjustment was done to BP, SBP was significantly different between genotype classes in hypertensives. BP was significantly lowered (p < 0.001) in normotensives (124.0 ± 17.6 / 74.1 ± 11.2 mmHg) as compared to hypertensives (150.3 ± 21.9 / 84.3 ± 12.6 mmHg).

**Table 4:** Blood pressure and other variables according to AGT genotypes in normotensive and hypertensive subjects. Values are expressed as mean. Values in parentheses represent standard deviation unless stated otherwise.

**Variables**	**Hypertensives**				**Normotensives**			
	
	**M/M (n = 33)**	**M/T (n = 46)**	**T/T (n = 22)**	p*	**M/M (n = 59)**	**M/T (n = 18)**	**T/T (n = 10)**	p*
**Age**	55.5 (8.9)	55.1 (9.0)	52.7 (9.0)	0.498	48.2 (10.2)	53.7 (9.0)	48.5 (12.4)	0.136

**SBP, mmHg**	151.5 (16.2)	150.8 (25.9)	147.6 (20.6)	0.795	122.0 (18.0)	129.1 (15.8)	126.9 (17.8)	0.283

**Adjusted SBP, mmHg**	150.9 (3.7)	149.6 (3.1)	150.8 (4.6)	0.014^ψ^	122.6 (1.9)	126.5 (3.5)	128.1 (4.5)	0.123^ψ^

**DBP, mmHg**	81.7 (14.7)	85.4 (12.3)	85.9 (9.3)	0.352	74.4 (12.0)	73.7 (11.4)	73.0 (4.6)	0.930

**Adjusted DBP, mmHg**	81.6 (2.2)	85.1 (1.9)	86.7 (2.8)	0.382^ψ^	74.4 (1.4)	73.0 (2.6)	73.8 (3.4)	0.106^ψ^

**BMI**	27.9 (4.7)	30.0 (4.9)	27.6 (6.3)	0.098	25.9 (3.4)	25.7 (3.3)	25.3 (4.9)	0.860

*Significant values were obtained through one-way ANOVA unless stated otherwise.

^ψ^Significant values were obtained through ANOVA using a general linear model with adjustment for age, sex, race and BMI.

By multiple regression analysis, age was only the predictor of SBP in the hypertensive group (P < 0.001) but not AGT genotype (R^2 ^= 0.147, p = 0.019 for SBP, R^2 ^= 0.064, p = 0.380 for DBP). BMI was the predictor for both SBP and DBP in the control group (P = 0.037 for SBP, P < 0.001 for DBP). Meanwhile, age and sex (P < 0.001 and 0.027 respectively) were the predictors for SBP in the control group (R^2 ^= 0.376, p < 0.001 for SBP, R^2 ^= 0.104, p = 0.172 for DBP). Sex, age, BMI, total cholesterol and Plasma Renin Activity and AGT genotype were all not predictors for SBP and DBP in hypertensive subjects (R^2 ^= 0.147, p < 0.019 for SBP, R^2 ^= 0.064, p = 0.380 for DBP).

### Associated risk factors

Although normotensive subjects were younger, there was no significant difference between the two groups with respect to vegetarian practices, exercise, smoking practices, alcohol drinking, glucose level (6.2 ± 2.7 mmol/L for cases and 6.7 ± 3.3 mmol/L for control, *p *= 0.198) TG (1.7 ± 0.9 mmol/L for cases and 1.6 ± 1.1 mmol/L for control, *p *= 0.558), TTLC (5.5 ± 1.1 mmol/L for cases and 5.3 ± 1.1 mmol/L for control, *p *= 0.084), LDLC (3.5 ± 1.0 mmol/L for cases and 3.3 ± 0.9 mmol/L for control, *p *= 0.162), HDLC (1.3 ± 0.5 mmol/L for cases and 1.3 ± 0.3 mmol/L for control, *p *= 0.875) and plasma sodium (139 ± 5 mmol/L for cases and 139 ± 3 mmol/L for control, *p *= 0.285) and potassium levels (4.0 ± 0.5 mmol/L for cases and 4.0 ± 0.4 mmol/L for control, *p *= 0.921).

However, using the Mann-Whitney U test shows that coffee consumption habit (69 cases and 71 controls, *p *= 0.038) whereas Student's T-test shows that BMI (28.8 ± 5.2 kg/m^2 ^for cases and 25.8 ± 3.6 kg/m^2 ^for control, *p *= 0.001) were lower in control subjects. The Pearson's Correlation test showed no correlation between exercise frequency and PRA in both hypertensives (*p *= 0.369, r = -0.098) and normotensives (*p *= 0.088, r = -0.170). No association was observed between years of smoking and years of drinking habits with PRA in both hypertensives (*p *= 0.994, r = -0.007 and *p *= 0.691, r = 0.040 respectively) and normotensives (*p *= 0.223, r = -0.123 and *p *= 0.174, r = -0.147 respectively).

## Discussion

In this study, it was found that the M235T polymorphism of the AGT gene is associated with essential hypertension. The relative risks for hypertension are 1.36 for subjects carrying the TT phenotype and 1.98 for those having allele T of the M235T variants. These results saw some agreement with some studies, but not with others. Jeunemaitre and associates [5] were the first to report the linkage of the molecular variants M235T with hypertension in the Whites/Caucasians. Subsequent studies among the Whites/Caucasians supported the former finding [16,17] while others did not [7,18,19]. The association studies in the Africans /African-Americans mostly found a negative association [8,13,20], but the T allele is associated with increased plasma AGT [13,20]. Studies on other Asian populations like the three studies in the Japanese population, found a positive association. [9,10,21] but not in others [14,22]. The Chinese [23] and the Taiwanese [11] population reported a positive association.

The frequency of the T235 variant among hypertensives this study, which was 0.45, is similar to the French cohort by the initial report by Jeunemaitre and associates [5]. Subsequent studies of the White populations reported frequencies of T235 allele in control groups of approximate 0.40, with the range from 0.31 [24] to 0.49 [7]. The frequency of T235 allele among Africans and African Americans is much higher than in whites, with the frequency as high as 0.92 [25]. Among the Japanese, the frequency is similarly high, around 0.75 [21]. Our study showed a higher Odds Ratio (O.R.) of 1.98 (95% CI, 1.46–2.67) compared to a recent meta-analysis [26] of 12 studies in the Whites which indicated that T235 is associated with a 20% increase risk of hypertension (O.R. = 1.22, 95% CI, 1.10–1.29).

In this study, the significantly higher prevalence of T allele in hypertensive females is in agreement with the study by Jeunemaitre *et al*., [5] which reported that the T allele was significantly more prevalent among female hypertensives (0.51) than in controls (0.37) (χ^2 ^= 16.9, p < 0.001). In contrast, Freire and associates [27], found that the AGT M235T homozygous mutation genotype was significantly higher in male compared to female, and Pereira *et al*. [28] reported that no association between gender and T allele in a cross-sectional study involving 647 females and 776 males. However, both of the studies were not confined to essential hypertensive patients.

Although the T235 allele is associated with increased plasma AGT in Blacks [20,25], the measurement of plasma AGT was not conducted in this study. However, the Plasma Renin Activity (PRA) test, which is the indicator of hyperaldosteronism, was carried out. Decreased PRA indicates primary hyperaldosteronism (adrenal-origin), while increased PRA indicates secondary hyperaldosteronism, an extra-adrenal cause. The concentration of AGT in blood is rate-limiting, and a change in its concentration can affect PRA [29]. The normal circulation level of AGT is close to Km, which means that a rise in plasma AGT could cause close to linear increase in the rate of angiotensin formation [29]. However, *in vivo*, an increase in plasma AGT will not increase the rate of angiotensin production, since the secretion of renin normally has a feedback relationship with angiotensin II so as to maintain a physiologically appropriate rate of formation of angiotensin II. [30] Exceptions of this condition are in individuals under estrogen influence [31] and in Cushing's syndrome [32]. An exclusively AGT-dependent hypertension is thus theoretically impossible, although two exceptional cases of hypertension associated with hepatic cell tumors producing large amounts of AGT have been reported [33,34]. Thus, this justifies that the measurement of PRA being done, instead of plasma AGT levels. PRA has an advantage of estimating the extent of aldosterone in response to the activation of the Renin-Angiotensin System (RAS). In this study, the PRA was significantly higher in hypertensive subjects (P < 0.0001), thus suggesting that the over activity of the RAS as a whole, thus contributing to hypertension. However, the PRA was not significantly different between groups of genotypes (P = 0.118).

There are several possible reasons for the discrepancies found between previous studies and this study. It might be due to ethnic differences due to the heterogenous population or sampling bias. The background of the study subjects recruited from a hypertensive clinic in this study might differ from that of subjects selected from the general population. Racial differences, including diverse social and cultural factors may have contributed to the different results. Discrepancies may also be related to different methodologies and study designs used.

The mechanism by which the molecular variant M235T of the AGT gene is related to hypertension is poorly understood. The AGT 235T variant has been found to be in complete linkage disequilibrium with a guanine-to-adenosine transition at -6 bp upstream of the initiation site of transcription [35]. *In vitro *tests of promoter activity and DNA-binding studies with nuclear proteins show that this nucleotide substitution affects the basal transcription rate of this gene in various cell lines, thereby the AGT T235 variant and increased plasma AGT levels [5] and hence might contribute to the elevation of blood pressure.

There were some limitations in this study. First, the normotensive subjects were relatively young compared with the hypertensives, although a 5-year difference in age might not have caused significant blood pressure variation. Secondly, the case-control design used to investigate the influence of the AGT 235T variant is known to be prone to selection bias and confounding especially when applied to the investigation of complex genetic traits, such as BP. The population studied was also not homogenous, as Malaysians consists of different ethnic groups, with different genetic make-up. While a positive association does not necessarily prove a causal relationship, it can provide useful information regarding the clinical importance of a genetic marker. Therefore, linkage and association studies are complementary, each providing a different type of information [36].

The mechanism by which the M235T variant contributes to the pathogenesis of hypertension needs to be elucidated further. It is also not possible to determine at the present whether the observed molecular variants of the AGT gene directly affect angiotensinogen function or whether they are markers of functional variants that have not yet been detected. If indeed the T235 variant directly affects plasma angiotensinogen concentration, it will be necessary to look for a possible difference in clearance rate of Km for renin between the two angiotensinogen isoforms. The response to antihypertensive agents, especially those blocking the RAS will have to be evaluated in patients classified according to their AGT genotype (pharmacogenomics). With the establishment of the association of the M235T variant with EH, further association studies of other cardiovascular diseases and diabetes involving this variant or other candidate genes involving the RAS system can be done. Hence, further genotyping of the Malaysian population could predict the risk of getting hypertension or other related cardiovascular disease.

## Conclusion

In this study, the T235 variant of the AGT gene is associated with essential hypertension in Malaysian subjects. The T235 variant is a risk factor or possibly a potential genetics marker for hypertension. Plasma renin activity is also significantly higher in hypertensive subjects but was not significantly different between groups of genotypes.

## Competing interests

The author(s) declare that they have no competing interests.

## Authors' contributions

YHS carried out the radioimmunoassay. Both YHS and KHL carried out the mutation studies, statistical analyses, developing questionnaires and drafted the manuscript. Both GD and SI carried out the anthropometry measurements and reviewed the patients' history and making decision regarding patient eligibility in the study. RR conceived the study, and participated in its design and coordination. All authors read and approved the final manuscript.

## Pre-publication history

The pre-publication history for this paper can be accessed here:


